# Diagnostic Accuracy of the National Library of Medicine (NLM) Screener Application in Screening for Malaria Parasites Among Blood Donors at the Korle‐Bu Blood Bank, Ghana: A Cross‐Sectional Study

**DOI:** 10.1155/jotm/8838042

**Published:** 2026-01-16

**Authors:** Samuel Bright Appiah, Samuel Osei, Nii Kpakpo Brown, Linda Eva Amoah, Ewurama Dedea Ampadu Owusu

**Affiliations:** ^1^ Department of Medical Laboratory Sciences, School of Biomedical and Allied Health Sciences, College of Health Sciences, University of Ghana, Legon, Accra, Ghana, ug.edu.gh; ^2^ Department of Biology, The University of Texas at Arlington, Arlington, Texas, USA, uta.edu; ^3^ Korle-Bu Teaching Hospital, Korle-Bu, Accra, Ghana, kbth.gov.gh; ^4^ Noguchi Memorial Institute of Medical Research, University of Ghana, Legon, Accra, Ghana, ug.edu.gh

**Keywords:** automated microscopy, blood bank, blood donors, malaria, mobile applications

## Abstract

**Background and Aims:**

Malaria is a deadly disease spread through the bite of an infected female *Anopheles* mosquito and remains the leading cause of mortality globally. Screening donated blood for malaria parasites is essential to prevent its transmission; however, conventional methods have limitations in accuracy and sensitivity. The National Library of Medicine (NLM) mobile application that uses machine learning algorithms to detect malaria parasites in blood smears could reduce some of these limitations. Hence, its performance in different settings needs to be evaluated. This study aimed to evaluate the performance of the NLM screener in screening malaria parasites against microscopy, rapid diagnostic tests (RDTs), and polymerase chain reaction (PCR) among blood donors at the Korle‐Bu Teaching Hospital (KBTH) in Accra, Ghana.

**Methods:**

We conducted a cross‐sectional study of 300 blood donors at the KBTH in Ghana. Each donor sample was tested with PCR (reference standard), microscopy, RDT, and the NLM screener app. Sensitivity, specificity, positive predictive value (PPV), negative predictive value (NPV), and Cohen’s *κ* were calculated with 95% confidence intervals (CIs). Agreement and paired outcomes were assessed with McNemar’s exact test.

**Results:**

PCR identified *Plasmodium falciparum* in 18/300 donors (6.0%). The NLM screener app showed sensitivity of 38.9% (7/18; 95% CI: 20.3–61.4), specificity of 60.6% (171/282; 95% CI: 54.8–66.2), PPV of 5.9% (7/118; 95% CI: 2.9–11.7), and NPV of 94.0% (171/182; 95% CI: 89.5–96.6), with negligible agreement (*κ* = −0.001). RDT and microscopy had lower sensitivities (44.4% and 27.8%, respectively) but perfect specificity (100%).

**Conclusion:**

The NLM screener app demonstrated low diagnostic performance in this setting. Applied to our donor pool, it would have led to 111 unnecessary discards (false positives) and 11 missed infections (false negatives). While promising, the app requires substantial improvement and validation before consideration for clinical use in transfusion safety programs.

## 1. Introduction

Malaria is a severe disease, which continues to be the primary cause of death worldwide [1]. Globally, the efforts of affected countries target early diagnosis and prompt treatment [2]. The burden of malaria is felt greatest in Africa with 11 countries contributing to two‐thirds of the world’s prevalence. Inadequate diagnostic systems in this continent have been observed to play a major role [3]. Of the 5 *Plasmodium* species that cause malaria in humans, *Plasmodium falciparum* is the most common, particularly in the World Health Organization (WHO) Africa area, accounting for 99.7% of malaria cases [4]. *Plasmodium* parasites are mostly transmitted to humans through the bite of infected female *Anopheles* mosquitoes; however, transfusion‐transmitted malaria (TTM) has posed a significant issue in both clinical and public health settings dating back to at least 1911 [5, 6].

In diagnosing malaria, microscopy, rapid diagnostic tests (RDTs), and polymerase chain reaction (PCR) are used, each with varying degrees of sensitivity and specificity [7, 8]. While RDTs are simple to use, do not require much skill, and have relatively short time to results, microscopy is time‐consuming and requires expert skills in microscopy. Additionally, the level of experience and expertise of the microscopist determines the degree of accuracy of the results [7–9]. In seeking to address some of these challenges, there have been attempts to automate image analysis and acquisition for the microscopic inspection of blood smears [10]. Light microscopy automation approaches that utilize deep learning and artificial intelligence techniques for digital image analysis are promising options, they require less skilled personnel and less supervision and offer fast and affordable diagnosis [9]. These include specially designed portable slide scanner that automatically gathers and evaluates focus stacks of blood smear pictures which was proposed by Gopakumar et al. in Germany in 2018 [11]. Also, a system that combines a large‐field‐of‐view, low‐resolution objective lens with a programmable light‐emitting diode (LED) array to enable the simultaneous capturing of hundreds of cells in a single image has been developed [12].

Although these approaches have a lot of promise, their complex hardware design makes it difficult to test them widely, especially in environments with limited resources [10]. Already available on the market is an automated microscope solution miLab which can detect malaria parasites, and has been evaluated in malaria endemic countries such as Sudan [13], Malawi [14], and Ghana and Ethiopia [15].

More recently, an artificial intelligence–based image analysis tool called the National Library of Medicine (NLM) screener has demonstrated promise in identifying malaria parasites in clinical samples, such as blood smears [16]. This mobile application consists of three modules: an image acquisition module, a parasite detection module that analyzes the field of view of a microscope to find malaria parasites, and a data management module that exports and preserves diagnostic records [16]. The NLM screener can potentially improve the accuracy and efficiency of malaria parasite screening in blood donors in the sense that it could reduce the need for expert malaria microscopists, who are scarce for parasite detection.

Despite progress in malaria diagnostics, there remains a critical gap in tools that can reliably and efficiently detect malaria parasites in donor blood, particularly in endemic regions. Existing methods such as microscopy and RDTs, though widely used, are limited by either operator expertise, time requirements, or variable accuracy. Studies have shown that diagnostic inaccuracies can contribute to malaria misdiagnosis and inappropriate treatment, further driving the risk of antimalarial resistance [17]. Automated digital tools such as the NLM screener app have demonstrated potential to overcome some of these limitations by providing rapid, accessible, and less skill‐dependent diagnostics in some settings and populations in Sudan [16] and Bangladesh [10]. However, few studies have evaluated their performance specifically in the context of blood donor screening in sub‐Saharan Africa, where TTM remains a significant yet underaddressed risk.

This study, therefore, aimed to evaluate the diagnostic accuracy of the NLM screener app compared to microscopy, RDT, and PCR among blood donors at the Korle‐Bu Teaching Hospital (KBTH) Blood Bank in Ghana.

## 2. Methodology

### 2.1. Study Design and Setting

This was a cross‐sectional diagnostic accuracy study conducted at the KBTH Blood Bank in Accra, Ghana. The hospital serves as the largest tertiary referral center in the country, making it a critical site for assessing transfusion safety in a malaria‐endemic region.

#### 2.1.1. Study Population

The study population comprised voluntary blood donors presenting at the Korle‐Bu Blood Bank during the study period. Eligible participants were individuals aged 18–60 years who had been clinically assessed and deemed fit to donate blood according to the National Blood Service criteria [18]. This criterialist thatblood donors should be between the age of 17–60 years, weigh at least 50 kg, and must be in good health. Donors with febrile illness at the time of donation, known history of malaria treatment within the past four weeks, or those who declined consent were excluded.

### 2.2. Sampling Method

The convenience sampling method was used for this study, where blood donors who were available and willing at the time of sample collection were enrolled as participants.

### 2.3. Data Collection Procedures

After informed consent, 4 mL of blood was collected from the sampling pouch of the blood donors who met the inclusion criteria and dispensed into ethylenediaminetetraacetic acid (EDTA) containers and mixed. A 50‐μL aliquot of blood was pipetted immediately from the EDTA tube onto the filter paper, the remainder was used for the malaria RDT test and blood film preparation. To make the dried blood spot (DBS), blood was applied onto a designated area to make a circle with a diameter of 1.2 cm on the Whatman No. 1 filter paper (dry blood spot card). The blood spot was allowed to air dry completely at room temperature and sealed in a plastic bag until it was processed for deoxyribonucleic acid (DNA) extraction and PCR.

### 2.4. Light Microscopy

Light microscopy examination was done to identify malaria species and diagnose malaria. A thick blood film was prepared according to the WHO standard procedures by transferring and spreading 6 μL of whole blood over a 1‐cm area on a clean and grease‐free microscope glass slide (CDC, 2016). The film was air‐dried, then stained with 10% dilute Giemsa stain for 10 min, carefully washed in tap water, and allowed to dry on a drying rack [19]. The film was observed by a WHO‐certified microscopist under the microscope at x40 for focusing and x100 objective oil immersion for the identification of malaria parasites [20].

### 2.5. NLM Screener Application

**Figure 1:**
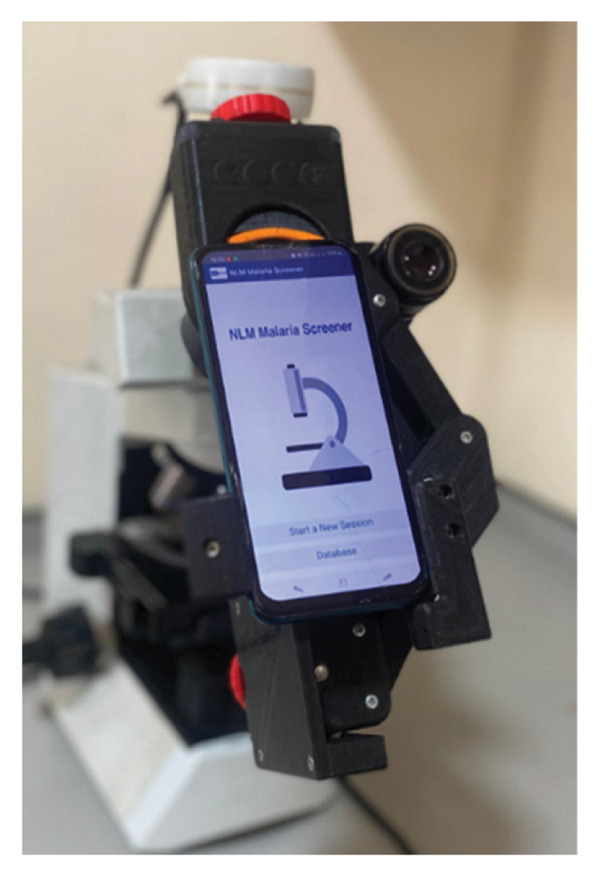
A setup of the NLM app on the phone affixed to the microscope.

The process of malaria screener–assisted microscopy requires attaching a smartphone atop a microscope’s ocular lens for malaria parasite identification according to developer instructions [16]. No prior training is required to use the app. The NLM screener application was downloaded from the Google Play Store and opened on a Samsung Galaxy J6 smartphone. A prepared glass slide of thick film was placed on the microscope stage with the focus and magnification of the microscope adjusted to a magnification of × 40. The smartphone camera lens was held close to the eyepiece of the microscope and aligned to capture the image of the slide. The application’s camera calibration feature was used to ensure that the captured image was properly aligned and calibrated. Brightness, contrast, and other settings in the application were adjusted to optimize the image quality. Annotation tools of the app were used to mark or highlight specific areas of interest in the image. The diagnostic results were saved, and the diagnosis and the captured image data were then later exported to an external database. An image of the setup is shown in Figure 1.

### 2.6. DNA Extraction and Nested PCR

DNA was extracted and quantified from a DBS card using a QIAamp DNA extraction kit following the manufacturer’s instructions (Qiagen, Germany) [21]. Nested PCR was done in a Veriti thermocycler to identify *Plasmodium* parasite species using already published *Plasmodium* primers rplu1 and rplu2 [16, 22]. Gel electrophoresis and visualization were performed to determine the size of DNA fragments based on their migration distance relative to the DNA ladder/marker bands.

### 2.7. Data Analysis

Data obtained from the study were entered into Microsoft Excel and analyzed using the Statistical Package for Social Sciences (SPSS) Version 28.0.1, where significant differences between diagnostic tools were determined by the nonparametric McNemar’s test using a *p* value of ≤ 0.5. Tests were two‐tailed. Agreement between the diagnostic tools was calculated using kappa coefficients. The following criteria were used: moderate agreement when kappa coefficient is 0.4–0.6, substantial agreement when 0.6–0.8, and excellent agreement when > 0.8 [23].

## 3. Results

### 3.1. Demographic Information of Study Participants

This study analyzed blood samples of 300 participants, comprising 249 males and 51 females. Overall, the sample was predominantly male and skewed toward younger adults, particularly those aged 18–40 years. This demographic profile provides context for interpreting diagnostic performance and malaria prevalence across age and sex groups (see Table 1).

**Table 1:** Demographic characteristics of study participants by age and sex.

Sex	Age range (yrs)	Frequency (%)	Mean ± SD (years)
Females (*n* = 51; 17%)	18–30	17/300 (5.7)	24 ± 3
	31–40	29/300 (9.7)	35 ± 2
	41–50	17/300 (5.7)	43 ± 10
	51–60	5/300 (1.7)	48 ± 4

Males (*n* = 249; 83%)	18–30	99/300 (33.0)	25 ± 4
	31–40	100/300 (33.3)	35 ± 3
	41–50	40/300 (13.3)	44 ± 2
	51–60	10/300 (3.3)	54 ± 2

### 3.2. Comparison of Diagnostic Tools Against PCR as Reference Standard

The frequency of true positives, false negatives (FNs), false positives (FPs), and true negatives for each method compared with PCR is shown in Table 2. The NLM app produced a high number of FPs, with none from RDT and microscopy. However, all three diagnostic tests had similar number of FNs with slight difference in NLM. Consequently, the sensitivity and specificity of NLM were both low at 43.8 (23.1–66.8) and 60.2 (54.4–65.8), respectively.

**Table 2:** Diagnostic performance of the NLM screener app, microscopy, and RDT compared with PCR (reference standard).

Test method	TP	FP	FN	TN	Sensitivity % (95% CI)	Specificity % (95% CI)	PPV %	NPV %	Accuracy % (95% CI)
NLM screener app	7	111	9	168	43.8 (23.1–66.8)	60.2 (54.4–65.8)	5.9	94.9	58.3 (52.7–63.8)
Microscopy	5	0	10	282	33.3 (15.2–58.3)	100.0 (98.7–100)	100.0	96.6	95.7 (92.7–97.5)
RDT	8	0	10	282	44.4 (24.6–66.3)	100.0 (98.7–100)	100.0	96.6	96.7 (94.0–98.2)

Abbreviations: FN, false negative; FP, false positive; TN, true negative; TP, true positive.

Table 3 presents a comparative analysis of the diagnostic performance of the NLM screener app, microscopy, and RDTs against PCR, the reference standard. The NLM screener app demonstrated slight agreement with PCR (*κ* = 0.09), indicating limited concordance and suggesting that its diagnostic decisions diverge significantly from the reference standard. Microscopy showed moderate agreement (*κ* = 0.47), reflecting a more reliable alignment with PCR results. However, RDT yielded the highest agreement (*κ* = 0.56), also in the moderate range, suggesting relatively strong consistency with PCR outcomes.

**Table 3:** Agreement and comparative diagnostic statistics for NLM screener app, microscopy, and RDT against PCR.

Test method	Cohen’s *κ* (95% CI)	McNemar’s test (*p* value)	Diagnostic odds ratio (95% CI)
NLM screener app	0.09 (slight agreement)	< 0.001	1.97 (0.79–4.92)
Microscopy	0.47 (moderate agreement)	0.25	35.2 (6.9–178.7)
RDT	0.56 (moderate agreement)	0.18	45.1 (9.1–223.5)

The NLM screener app had a statistically significant *p* value (< 0.001), indicating a significant difference in classification compared to PCR. But microscopy (*p* = 0.25) and RDT (*p* = 0.18) did not show significant differences, implying that their diagnostic classifications are statistically comparable to PCR.

With low DOR of 1.97 (95% confidence interval [CI]: 0.79–4.92), NLM screener app indicated poor discriminatory power between malaria‐infected and noninfected individuals, whereas microscopy and RDT demonstrated substantially higher DORs of 35.2 (6.9–178.7) and 45.1 (9.1–223.5), respectively, suggesting strong diagnostic performance and better ability to distinguish true positives from FPs.

Due to the fact that microscopy is the gold standard for malaria diagnosis, NLM and RDT results were compared with microscopy in Table 4. While RDT showed a slightly higher sensitivity, the overlapping CIs meant that the difference was not statistically significant. Specificity of RDT and NLM was also comparable.

**Table 4:** Comparison of diagnostic performance of the NLM screener app and RDT across different reference standards.

Test	Reference standard	Sensitivity % (95% CI)	Specificity % (95% CI)	Accuracy %	PPV %	NPV %
NLM screener app	PCR	43.8 (23.1–66.8)	60.2 (54.4–65.8)	58.3	5.9	94.9
	Microscopy	46.7 (21.3–73.4)	58.1 (52.2–63.7)	57.7	6.8	96.7

RDT	PCR	44.4 (24.6–66.3)	100.0 (98.7–100)	96.7	100.0	96.6
	Microscopy	53.3 (26.6–78.7)	100.0 (98.7–100)	97.3	81.8	97.9

## 4. Discussion

Accurate and timely diagnosis of malaria is critical for effective treatment and control, particularly in endemic regions. This study evaluated the diagnostic performance of the NLM screener app, comparing it to microscopy, RDTs, and PCR, providing insights into its utility in field settings, particularly for screening of donated blood.

In our study population, male donors were higher than female donors. This disparity is similar to observations in other countries in Europe [24], sub‐Saharan Africa [25], and Ghana [26], which has been attributed to cultural and social norms, biological differences such as menstruation and pregnancy affecting hemoglobin levels, and women being more prone to adverse reactions as a result [24, 27]. Nonetheless, this could also be due to the convenience sampling nature of the study which could have introduced selection bias and limited representativeness. While this could have influenced the malaria pattern observed in our study, preliminary subgroup analysis attempted on the low number of malaria positives showed sex was not a significant predictor of malaria positivity.

In using PCR as the reference standard for the detection of *P. falciparum*, sensitivity and specificity of the NLM app were low. These findings suggest that although the NLM app is accessible and easy to use, its current version is not suitable as a stand‐alone screening tool for blood donors. Our findings contrast with those of Yu et al. who reported 100% sensitivity and 51.1% specificity for the NLM app in symptomatic patients in Sudan [16]. The lower sensitivity observed in our study likely reflects differences in study populations; for instance, our donors were asymptomatic and may have had low‐density parasitemia, whereas their studies evaluated patients presenting with clinical malaria and were likely to have higher parasitemia. Low parasite densities are more difficult for both human and AI‐based microscopy to detect [7]. Differences in slide preparation, staining quality, and operator experience may also explain performance variation. Similarly, while image‐based tools have shown promise in Bangladesh and other settings [9], the variation across contexts highlights the importance of validating AI tools in diverse epidemiological and laboratory environments. Automated image‐based diagnostics like the NLM screener remain attractive because they are scalable, low‐cost, and less dependent on limited expert microscopists in endemic regions like Africa [28–30]. However, their success hinges on image quality and algorithm performance. Improvements in slide preparation, camera alignment, and algorithm training with diverse datasets are necessary to reduce misclassification. Our choice of PCR as the reference standard was appropriate given its high sensitivity, though it may partly explain the lower apparent sensitivity of the index tests compared to studies using microscopy alone. Nevertheless, overlapping CIs between reference standards [31] suggest comparability.

The NLM app generated a high number of FPs (*n* = 111), similar to a study conducted by Yu et al. [16] in two primary‐level hospitals in Sudan. This can be attributed to misclassification of artifacts, staining debris, or poor image alignment, as previously noted in other image‐based diagnostic studies [7, 16]. Potentially serious implications for transfusion services can arise as a result. To put this in context, from our study sample of 300 donors, 111 units (37%) would have been unnecessarily discarded, further straining Ghana’s already inadequate blood supply. The country’s Blood Collection Index (BCI) has consistently remained below the WHO‐recommended threshold of 10 units per 1000 population, with reported values of 5.6 in 2017, 6.0 in 2019, and 5.7 in 2021 [32, 33]. Adoption of the NLM app in its current form could therefore worsen shortages by further reducing available safe blood.

Equally concerning are the 11 FNs identified, representing infected units that would have been transfused undetected. This could have been due to low parasite densities which were mainly between 1000 and 2000 parasites/μL in our study.

The consequences of TTM range from uncomplicated malaria to severe or cerebral disease, particularly in vulnerable groups such as children and pregnant women [34, 35]. During emergencies such as trauma, serious accidents, surgery, and gynecological hemorrhages, blood transfusions are crucial to saving lives [36]. The implications of transfusion of *Plasmodium*‐infected blood range from uncomplicated malaria in the recipient to severe/complicated or cerebral malaria. While existing partial immunity may provide some protection from severe symptoms in some recipients who live in malaria‐endemic areas [33, 35], severe or cerebral malaria infection could be fatal in the vulnerable populations such as children and pregnant women [34].

In contrast, while the RDT and microscopy did not generate FPs, they missed several low‐density infections, underscoring the persistent limitations of existing donor screening methods. The burden of asymptomatic carriage underscores the need for effective donor screening. Yet, routine malaria screening of donor blood is not performed in Ghana or most sub‐Saharan countries [37–39]. By contrast, nonendemic regions such as the USA and EU/EEA adopt strict deferral policies, excluding donors with prior malaria exposure or residence in endemic areas [40]. Encouragingly, Ghana’s National Malaria Elimination Strategic Plan 2024–2028 proposes integrating malaria into donor blood screening guidelines [32].

Several factors may have contributed to the lower performance of the NLM screener in detecting malaria parasites. One significant factor could have been the quality and resolution of the images analyzed by the screener. Since the app relies on AI algorithms to analyze these images, any degradation in image quality, such as low resolution or the presence of artifacts in the blood smear, could hinder the screener’s ability to accurately identify parasites, affecting both sensitivity and specificity. Additionally, real‐world scenarios often present inherent variability, including the presence of artifacts in blood films [16], which may stem from poor preparation techniques [7]. These factors could have further challenged the algorithm’s ability to distinguish true positive and true negative cases accurately. Moreover, the default setting (classifier sensitivity level default value of DL:50) of the app was used throughout the study. All of the above may have contributed to the performance of the app. Achieving a balance between sensitivity and specificity is crucial for the effectiveness of the NLM app, underscoring the necessity for continuous refinement and optimization of the application to address these challenges and improve its diagnostic accuracy in diverse clinical settings.

The decision to use highly sensitive PCR as the reference standard in evaluating the performance of the NLM app could also have impacted the perceived performance of the NLM app. PCR is known for its ability to detect very low parasite densities, making it a stringent reference standard. However, in this study, the sensitivity and specificity of the NLM app, using PCR as reference, were found to be comparable when microscopy was used as a reference standard, with overlapping CIs. This suggests that the difference in performance between the two reference standards may not be significant [31]. Interestingly, both malaria RDT and microscopy showed generally low sensitivity in the study, further highlighting the complexities in accurately diagnosing malaria across different diagnostic methods. Overall, these findings underscore the need for careful consideration of reference standards and continued evaluation of diagnostic methods to ensure their effectiveness in real‐world clinical settings.

This study has notable strengths, including the comparison of the novel test with three existing diagnostic tests (microscopy, RDT, and PCR) that are available in malaria‐endemic regions, be it at the primary, secondary, or tertiary healthcare level. Additionally, the relatively large sample size and evaluation in a high‐volume blood national blood bank provide a representative context for the population. However, a study limitation such as the use of convenience sampling could have affected its representativeness. Secondly, the male‐dominated donor pool can reduce generalizability of findings, but this could not be accurately corroborated due to the inability to accurately perform subgroup analyses by sex or age on account of small number of positives. Moreover, slide quality and operator variability may also have influenced the NLM app performance.

## 5. Conclusion

Although the NLM malaria screener app offers rapid and low‐cost diagnostics, its current sensitivity (38.9%) and specificity (60.6%) fall below acceptable thresholds for blood donor screening. Applied to our cohort, the app would have resulted in substantial misclassification, including the unnecessary discard of 111 blood units and 11 missed infections—outcomes that could exacerbate Ghana’s already strained blood supply. Thus, significant improvements in algorithm accuracy, image capture, and multicenter validation are necessary to achieve maximum clinical use. Until then, conventional methods such as microscopy, RDTs, and PCR remain essential for ensuring transfusion safety in malaria‐endemic settings.

## Ethics Statement

Ethical approval was sought from the Ethical and Protocol Review Committee of the School of Biomedical and Allied Health Sciences (SBAHS) of the College of Health Sciences of the University of Ghana with Ethics Identification Number: SBAHS/AA/MLAB/10710149/2022‐2023. Approval to carry out the study was obtained from the Head of Research at the National Blood Bank and Korle‐Bu Teaching Hospital Blood Bank. Informed consent was sought from the participants before their recruitment for the study after detailing the risks and benefits of participation and assuring their confidentiality.

## Disclosure

All authors have read and approved the final version of the manuscript. The lead author, Ewurama Dedea Ampadu Owusu, affirms that this manuscript is an honest, accurate, and transparent account of the study being reported; that no important aspects of the study have been omitted; and that any discrepancies from the study as planned (and, if relevant, registered) have been explained.

## Conflicts of Interest

The authors declare no conflicts of interest.

## Author Contributions

Ewurama Dedea Ampadu Owusu and Samuel Bright Appiah conceived the study. Ewurama Dedea Ampadu Owusu, Samuel Bright Appiah, Samuel Osei, and Linda Eva Amoah designed the methodology. Samuel Bright Appiah and Nii Kpakpo Brown collected the data. Samuel Bright Appiah, Nii Kpakpo Brown, and Samuel Osei contributed data or analysis tools. Samuel Bright Appiah, Nii Kpakpo Brown, Samuel Osei, Linda Eva Amoah, and Ewurama Dedea Ampadu Owusu performed the analysis. Samuel Osei and Ewurama Dedea Ampadu Owusu drafted the paper. All authors reviewed the paper.

Ewurama Dedea Ampadu Owusu had full access to all of the data in this study and takes complete responsibility for the integrity of the data and the accuracy of the data analysis.

Samuel Bright Appiah and Samuel Osei are co‐first authors.

## Funding

No funding was received for this manuscript.

## Data Availability

Additional data are available upon request to the corresponding author.
